# A Higher Skeletal Muscle Mass and Lower Adiposity Phenotype Is Associated with Better Cardiometabolic Control in Adults with Hip and Knee Osteoarthritis: Results from the Chilean National Health Survey 2016–2017

**DOI:** 10.3390/nu15194263

**Published:** 2023-10-05

**Authors:** Francisco Guede-Rojas, Paulina Ibacache-Saavedra, María Inés Leal, Marcelo Tuesta, Cristóbal Durán-Marín, Fernanda Carrasco-Marín, Igor Cigarroa, Cristian Alvarez, Mikel Izquierdo, Pedro Delgado-Floody

**Affiliations:** 1 Exercise and Rehabilitation Sciences Institute, School of Physical Therapy, Faculty of Rehabilitation Sciences, Universidad Andres Bello, Santiago 7591538, Chile; francisco.guede@unab.cl (F.G.-R.); paulina.ibacache@unab.cl (P.I.-S.); maria.leal@unab.cl (M.I.L.); marcelo.tuesta@unab.cl (M.T.); cristian.alvarez@unab.cl (C.A.); 2 Physical Therapy, Faculty of Rehabilitation Sciences Carrera de Kinesiología, Universidad Andres Bello, Concepción 4260000, Chile; caduranmarin@gmail.com; 3 Centro de Vida Saludable, Universidad de Concepción, Concepción 4030000, Chile; fercarrasco@udec.cl; 4 Escuela de Kinesiología, Facultad de Salud, Universidad Santo Tomás, Los Ángeles 4440000, Chile; icigarroa@santotomas.cl; 5 Navarrabiomed, Hospital Universitario de Navarra (HUN), Universidad Pública de Navarra (UPNA), IdiSNA, 31006 Pamplona, Spain; mikel.izquierdo@gmail.com; 6 CIBER of Frailty and Healthy Aging (CIBERFES), Instituto de Salud Carlos III, 28220 Madrid, Spain; 7 Department of Physical Education, Sports and Recreation, Universidad de La Frontera, Temuco 4811230, Chile; 8 Department of Physical Education and Sports, Faculty of Sport Sciences, University of Granada, 18011 Granada, Spain

**Keywords:** osteoarthritis, diabetes, arterial hypertension, older adults, skeletal muscle mass, body fat

## Abstract

Objective: This study aimed to (1) characterize cardiometabolic factors in self-reported hip and knee osteoarthritis (OAD) across four body composition phenotypes defined by muscle mass and adiposity, and (2) associate risk factors with diabetes and hypertension (HTN). Methods: A cross-sectional analysis of the Chilean National Health Survey 2016–17 (*n* = 4996) stratified participants into four groups: low skeletal muscle mass/high waist circumference (Low-SMM/High-WC), low SMM/low WC (Low-SMM/Low-WC), high SMM/high WC (High-SMM/High-WC), and high SMM/low WC (reference group). Each group was further divided into subgroups with or without diagnosed hip or knee OAD. The main outcomes were fasting plasma glucose, systolic (SBP)/diastolic (DBP) blood pressure (continuous outcomes), and other secondary factors such as cardiovascular risk (CVR). Results: In the hip OAD subgroup, the Low-SMM/High-WC groups had significantly higher SBP versus the reference value (145 vs. 127 mmHg, *p* < 0.0001, diff +18 mmHg). In the knee OAD subgroup, the Low-SMM/High-WC groups had significantly higher SBP versus the reference value (141 vs. 134 mmHg, *p* < 0.0001, diff +7 mmHg). The SBP showed a significant interaction between the group and OAD diagnosis (*p* = 0.007 hip OAD; *p* < 0.0001 knee OAD). Conclusions: Hip and knee OAD associates with elevated SBP/DBP in older adults. OAD groups showed an OR above 2 for diabetes, 2.7 for HTN, 4.5 for metabolic syndrome, and over 2 for moderate-to-high cardiovascular risk. OAD interacts substantially with cardiometabolic factors, especially in low muscle mass/high adiposity phenotypes. Lifestyle optimization of physical activity and nutrition to preserve muscle mass and mitigate adiposity is essential for cardiometabolic health promotion in OAD patients.

## 1. Introduction

Osteoarthritis (OAD) is a chronic, disabling disease and a major global public health issue as a leading cause of disability worldwide [1]. The prevalence of OAD varies by age, gender and region, and is estimated to affect over 10% of the population, commonly affecting the knee and hip joints [2]. Traditionally, OAD has been characterized by chronic pain, inflammation, bone and cartilage changes, joint deterioration, and metabolic syndrome (MetS) [3].

Skeletal muscle mass (SMM) tends to decrease in physically inactive adults with OAD, promoting sarcopenia which is highly prevalent in OAD patients [4]. Lower extremity SMM is also an independent risk factor for developing OAD [5]. Additionally, obesity is a key risk factor for OAD onset and progression [6], since adipose tissue is an endocrine organ that secretes inflammatory adipokines implicated in cardiometabolic diseases [7].

Therefore, physical inactivity leads to reduced SMM (i.e., decreased fitness and function) and increased adiposity in OAD patients, conferring high cardiometabolic disease risk including obesity, diabetes, arterial hypertension (HTN), and MetS. These conditions predispose OAD patients to elevated cardiovascular risks such as myocardial infarction and stroke, as well as dyslipidemia (high total cholesterol [Tc], low-density lipids [LDL-c], high plasma triglycerides [Tg], and lower levels of high-density lipoproteins [HDL-c]), including glycemic dysregulation (high levels of fasting plasma glucose [FPG]). A recent meta-analysis showed that OAD patients have increased pulse wave velocity indicating endothelial dysfunction and atherosclerosis risk [8]. Our research also found that HTN patients exhibit a higher exertional heart rate and a reduced heart rate reserve using traditional theoretical maximum methods [9]. Thus, beyond managing OAD-related pain and inflammation, addressing cardiometabolic risk factors such as FPG and blood pressure is relevant in OAD to prevent diabetes, HTN, MetS, and major cardiovascular events.

Most OAD research has focused on either the hip or knee joint. However, analyzing both hip and knee OAD from a cardiometabolic phenotype perspective, using practical body composition outcomes such as calf circumference (CC) as an indicator of SMM and waist circumference (WC) as an indicator of adiposity would be interesting. For example, Pagoto et al. reported CC was a valid SMM marker in older adults [10]. Similarly, extensive evidence shows WC correlates with total body fat [11]. Thus, independent of lifestyle factors (nutrition, physical activity levels, tobacco, alcohol consumption, sleep, etc.), adults can express phenotypical adaptations in SMM and WC as high/low SMM and WC. It is well-established that lower SMM and higher adiposity are associated with greater diabetes and HTN risk in adults [12], and that lower SMM is also related to higher inflammation [13].

However, little Chilean evidence exists on diabetes and HTN risk in OAD adults with different body composition phenotypes (e.g., low SMM and high WC, high SMM and low WC, or other combinations). Considering that the accelerated aging process of the population has increased OAD prevalence in some countries [14], this study aimed to (1) characterize cardiometabolic risk factors in hip/knee OAD across four phenotypes based on CC and WC and (2) associate cardiometabolic risks with glucose/blood pressure levels. We hypothesized that adults with low SMM and high WC would show poorer glucose controls and blood pressure than those with high SMM and low WC.

## 2. Materials and Methods

This was a cross-sectional observational study based on the Chilean National health Survey 2016–17 (NHS16-17), which was a prevalence-based, multi-stage, and representative study applied at home and using random stratified-by-conglomerates methods. The data included a population with or without ethnic/foreign origin, from urban/rural areas of this country. The study only included members of the population who were registered in the public Chilean health system, and the time point of the study’s data collection was between 2015 and 2016. The study protocol was approved by the Ethical Committee of the Escuela de Medicina de la Pontificia Universidad Católica de Chile (16-019), and all participants signed an informed consent form [15]. All information, data, and reports of the NHS16-17 can be freely accessed at the Epidemiological Unit of the Chilean health Ministry http://epi.minsal.cl/encuesta-ens-descargable (accessed on 3 July 2023). Additional information based on the NHS16-17 can be found on the web page of the ELHOC Research Consortium–Epidemiology of Lifestyle and Health Outcomes in Chile at https://sites.google.com/view/elhoc/home?authuser=0 (accessed on 3 July 2023).

### 2.1. Participants

Considering the total NHS16-17 sample (*n* = 6233) of participants, the participant data were stratified by four different body composition phenotypes groups of low SMM and high WC (Low-SMM/High-WC), low SMM and low WC (Low-SMM/Low-WC), high SMM and high WC (High-SMM/High-WC), and high SMM and low WC (High-SMM/Low-WC); see below in the ‘Phenotypes’ section for definitions of high/low SMM and WC. Subsequently, the sample of each group was divided according to members of the population who reported diagnosed hip osteoarthritis (Hip-OAD), did not report diagnosed hip osteoarthritis (No-Hip-OAD), reported diagnosed knee osteoarthritis (Knee-OAD), or did not report diagnosed knee osteoarthritis (No-Knee-OAD).

**Figure 1:**
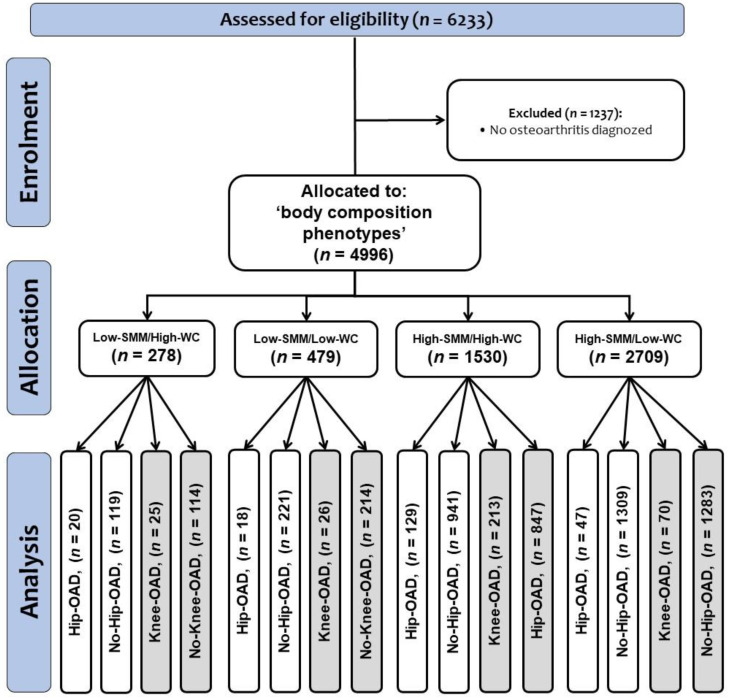
Study design. (OAD) Osteoarthritis diagnosed. Categories of osteoarthritis conditions are described as hip osteoarthritis diagnosed (Hip-OAD), no hip osteoarthritis diagnosed (No-Hip-OAD), knee osteoarthritis diagnosed (Knee-OAD), no knee osteoarthritis diagnosed (No-Knee-OAD).

Thus, final sample size according to each group and their categories was as follows: ‘Low’ SMM and ‘High’ WC (Low-SMM/High-WC, *n* = 278 [Hip-OAD, *n* = 20; No-Hip-OAD, *n* = 119] and [Knee-OAD, *n* = 25; No-Knee-OAD, *n* = 114]), ‘Low’ SMM and ‘Low’ WC (Low-SMM/Low-WC, *n* = 479 [Hip-OAD, *n* = 18; No-Hip-OAD, *n* = 221] and [Knee-OAD, *n* = 26; No-Knee-OAD, *n* = 214]), ‘High’ SMM and ‘High’ WC (High-SMM/High-WC, *n* = 1530 [Hip-OAD, *n* = 129; No-Hip-OAD, *n* = 941] and [Knee-OAD, *n* = 213; No-Knee-OAD, *n* = 847]), and ‘High’ SMM with ‘Low’ WC (High-SMM/Low-WC, *n* = 2709 [Hip-OAD, *n* = 47; No-Hip-OAD, *n* = 1309] and [Knee-OAD, *n* = 70; No-Knee-OAD, *n* = 1283]). The study design can be seen in Figure 1.

### 2.2. Phenotypes by Hip and Knee Osteoarthritis Diagnosed

The four different phenotypes were modelled using both CC (i.e., as a SMM marker) and WC (i.e., as an adiposity marker) using the NHS16-17 data. We used a cut-off point of 34 cm CC considering its major sensitivity (71.5%) and specificity (77.4%) for assessing SMM in Latin-American (i.e., Brazilian) adult men, and 33 cm for adult women, which has also been shown to have similar sensitivity (80.0%) and specificity (84.6%) [10].

The WC was categorized as high WC (men ≥ 90 cm, women ≥ 80 cm) or low WC (men < 89 cm, women < 79 cm), considering that these parameters are currently used in Chile [15]. An inextensible tape was used to measure both CC and WC.

### 2.3. Diabetes and Arterial Hypertension Markers (Main Outcomes)

To determine diabetes risk, FPG and glycated hemoglobin (HbA1c) were measured in fasting conditions (i.e., 8 h) by professional nurses, similar to a previous study [16]. Additionally, we reported the diabetes risk, using the question “diabetes suspects in fasting state” with categorical answers ‘Yes’ or ‘No’, which was included in the NHS16-17, where odds ratios (ORs) were reported (see statistical analysis section below).

To determine HTN risk, the systolic (SBP) and diastolic (DBP) blood pressure were measured in the left arm three times and the average of these attempts was registered. From here, we used the American Heart Association 2018 blood pressure categorization: ‘Normal BP’ was defined as SBP/DBP less than 120/80 mmHg, ‘elevated blood pressure’ as SBP/DBP between 120 and 129/80 mmHg, ‘stage 1 HTN’ as SBP/DBP between 130 and 139/80–89 mmHg, and ‘stage 2 HTN’ as SBP/DBP greater than or equal to 140/90 mmHg [17]. These measurements were carried out using an automatic monitor (OMRON^TM^, model HEM 7114, Tokio, Japan) similar to previous studies using the NHS16-17 data [18], and was applied by professional nurses in at-home conditions. Similar to diabetes risk, to report the risk for suffering of HTN, we also reported the OR statistical parameters, using the question “Arterial hypertension suspects” with categorical answers ‘Yes’ or ‘No’, which was included in the NHS16-17 (see statistical analysis section below).

### 2.4. Secondary Cardiometabolic Risk Factors (Secondary Outcomes)

As secondary outcomes, we included total cholesterol (Tc), low-density lipid cholesterol (LDL-c), high-density lipid cholesterol (HDL-c), and plasma triglycerides (Tg), which are classified according to the National Cholesterol Education Program NCEP ATP-III criteria [19]. As a ‘mineral content’ we included the 25-OH vitamin D2 + D3 outcome, while both gamma (GGT) and pyruvic glutamyl transferase (PGT), which are markers of non-alcoholic fatty liver disease, were also reported. Finally, we included the C-reactive protein as an ‘inflammation’ marker.

Weight was measured using a digital electronic scale OMRON^TM^ model HN 289 (OMRON Corporation, Tokio, Japan) with a sensitivity of 100 g and maximum weight capacity of ~150 kg. Height and waist circumference were measured using an inextensible tape, similar to previous studies [20]. The BMI was calculated using both weight and height following standard recommendations [21].

To determine physical activity (PA) levels, the Global Physical Activity Questionnaire v2 was used (GPAQv2), similar to a previous study of the Chilean population [18]. Thus, physical activity was described according to its intensity as PA of vigorous intensity (PA_VI_), PA of moderate intensity (PA_MI_), and PA of light intensity (PA_LI_), the latter corresponding with light activities such as walking, transport, or cycling. This information was obtained from the following questions included in the NHS16-17 as follows: (a) to measure the PA_VI_: on one of those days when you engage in ‘intense’ physical activities, how much time do you usually spend on those activities? (b) to report PA_MI_: on one of those days when you do “moderate” physical activities, how much time do you usually spend on those activities?; and (c) to report the PA_LI_: on a normal day, how much time do you spend walking or biking to get around?

To determine sleep time, the following categorization was used similar to a previous study: <7 h/day, from 7 to 9 h/day, and >9 h/day to denote low, middle, and high levels of sleep time, respectively [16].

### 2.5. Other Cardiovascular Risk Estimation

As additional information to the diabetes and HTN risk, we estimated the risk for suffering MetS, as well as a ‘moderate’ and ‘high’ cardiovascular risk (CVR). Thus, CVR was categorized using a punctuation scale with ‘low’ (0–4 points), ‘moderate’ (5 to 9 points), and ‘high’ (≥10 points) risk using the 5 metabolic syndrome outcomes (SBP/DBP blood pressure, FPG, HDL-c, and Tg), as well tobacco habit, alcohol consumption, dyslipidemia, and sleep patterns outcomes. This information was obtained from the following three questions included in the NHS16-17 as follows: (a) in the self-report on acute myocardial infarction “Has a doctor or physician ever told you had or suffered a heart attack?” The prevalence from those who answered “Yes” was used; (b) the question for the self-reported prevalence of stroke “Has a doctor or physician ever told you had or suffered a stroke?” or “had or suffered a stroke or cerebral thrombosis (or stroke)?”; and (c) the question about the self-reported prevalence of peripheral venous disease “Has a doctor or physician ever told you had or suffered from peripheral vascular disease or to the arteries in your legs?” [22]. In this study, only the risk of suffering ‘moderate’ and ‘high’ CVR were reported.

### 2.6. Statistical Analyses

Data for continuous outcomes are shown as mean and ± standard deviation (SD) in tables and as mean and 95% confidence interval (CI) in figures. For categorical outcomes, information is shown as frequencies (*n* = ) and percentages (%). The normality was tested using the Shapiro–Wilk test and using histograms and Q–Q plots. For continuous outcomes, the comparison of each main and secondary outcome among the four phenotype groups (Low-SMM/High-WC, Low-SMM/Low-WC, High-SMM/High-WC, High-SMM/Low-WC) by each hip and knee OAD category was applied using a univariant test, with the High-SMM/Low-WC group used as the reference (Ref.). Additionally, associations were tested using multinomial logistic regression (MLR), where the Wald Chi–square was registered along with the pseudo-McFadden R^2^ as predictive test for dependent outcomes. To the MLR, the (*n* = 4996) data participants that included both hip and knee OAD conditions were included. The following models were used: (Model 1) Include calf circumference [CC] ≤ 33.9 cm plus WC ≥ 90.0 cm for men, or CC ≤ 32.9 cm plus WC ≥ 80.0 cm for women, geographic area, region, age, body mass index, and sex. (Model 2) Include calf circumference [CC] ≤ 33.9 cm plus WC ≤ 89.9 cm for men, or CC ≤ 32.9 cm plus WC ≥ 80.0 cm for women, geographic area, region, age, body mass index, and sex. (Model 3) Include calf circumference [CC] ≥ 34.0 cm plus WC ≥ 90.0 cm for men, or CC ≤ 32.9 cm plus WC ≥ 80.0 cm for women, geographic area, region, age, body mass index, and sex. (Model 4) Include calf circumference [CC] ≥ 34.0 cm plus WC ≤ 89.9 cm for men, or CC ≤ 32.9 cm plus WC ≥ 80.0 cm for women, geographic area, region, age, body mass index, and sex. Model 4 was used as reference model. We also calculated the risk for suffering diabetes, HTN, MetS, and ‘moderate’ and ‘high’ CVR using OR tests and showing their 95% CI. These analyses were adjusted by geographic area, region, sex, age, and BMI. All statistical analyses were developed using the SPSS^TM^ software version 25 for Windows (IBM SPSS Inc., Chicago, IL, USA).

## 3. Results

### 3.1. Baseline Characteristics

In the present study, we used both CC (as an SMM indicator) and WC (as an adiposity indicator). The Receiver Operator Characteristic (ROC) was examined for WC (sensitivity 90.2%, and specificity 28.4% to 90 cm as cut-off point) for men and women (sensitivity 52.4%, and specificity 0.05% to 80 cm as cut-off point). The ROC was also determined for CC (sensitivity 84.0%, and specificity 36.2% to 34 cm as cut-off point) for men and women (sensitivity 74.3%, and specificity 26.6% to 33 cm as cut-off point).

The general characteristics of the groups with and without hip and knee osteoarthritis are shown in (Table 1 and Table 2).

### 3.2. Diabetes and Arterial Hypertension Markers (Main Outcomes)

**Figure 2:**
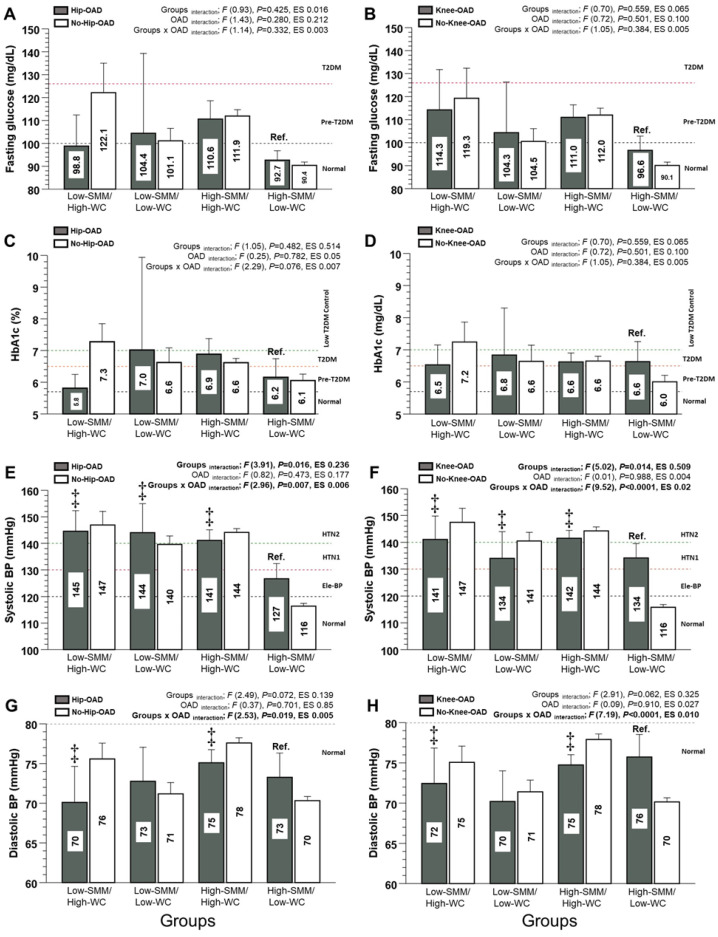
Fasting plasma glucose, glycated haemoglobin, systolic and diastolic blood pressure through four different human adult phenotypes with hip and knee osteoarthritis diagnosed (OAD). Groups are described as follows: low skeletal muscle mass and high waist circumference phenotypical model (Low-SMM/High-WC), low skeletal muscle mass and low waist circumference phenotypical model (Low-SMM/Low-WC), high skeletal muscle mass and high waist circumference phenotypical model (High-SMM/High-WC), and high skeletal muscle mass and low waist circumference phenotypical model (High-SMM/Low-WC), reference group (Ref.). Categories are described as follows: Hip osteoarthritis diagnosed (Hip-OAD), no hip osteoarthritis diagnosed (No-Hip-OAD), knee osteoarthritis diagnosed (Knee-OAD), no knee osteoarthritis diagnosed (No-Knee-OAD), type 2 diabetes mellitus (T2DM), prediabetes (Pre-T2DM), elevated blood pressure (Ele-BP), hypertension stage 1 (HTN1), and hypertension stage 2 (HTN2). (HbA1c) Glycated hemoglobin. (ES) Denotes Cohen *d* effect size. (‡) Denotes significant differences vs. Ref. group at *p* < 0.0001. (**A**) Fasting glucose in Hip and No-Hip-OAD. (**B**) Fasting glucose in Knee and No-Knee-OAD. (**C**) Glycated hemoglobin in Hip and No-Hip-OAD. (**D**) Glycated hemoglobin in Knee and No-Knee-OAD. (**E**) Systolic blood pressure in Hip and No-Hip-OAD. (**F**) Systolic blood pressure in Knee and No-Knee-OAD. (**G**) Diastolic blood pressure in Hip and No-Hip-OAD. (**H**) Diastolic blood pressure in Knee and No-Knee-OAD.

For FPG and HbA1c, none of the interactions tested were significant (Figure 2A–D).

For the SBP of the Hip-OAD category, there were significant differences between the Low-SMM/High-WC group and the Ref. group (145 and 95%CI [135; 153] vs. 127 [120; 132 mmHg], *p* < 0.0001, *diff*. +18 mmHg), the Low-SMM/Low-WC group and the Ref. group (144 [134; 154] vs. 127 [120; 132 mmHg], *p* < 0.0001, *diff*. +17 mmHg), and the High-SMM/High-WC group and the Ref. group (141 [137; 145] vs. 127 [120; 132 mmHg], *p* < 0.0001, *diff*. +14 mmHg), showing a significant interaction of the groups x OAD (*p* = 0.007) (Figure 2E). For the SBP of the Knee-OAD category, there were significant differences between the Low-SMM/High-WC group and the Ref. group (141 [133; 149] vs. 134 [129; 139 mmHg], *p* < 0.0001, *diff*. +7 mmHg), the Low-SMM/Low-WC group and the Ref. group (134 [126; 142] vs. 134 [129; 139 mmHg], *p* < 0.0001, *diff*. +0.9 mmHg), and the High-SMM/High-WC group and the Ref. group (142 [139; 144] vs. 134 [129; 139 mmHg], *p* < 0.0001, *diff*. +8 mmHg), showing a significant interaction of the groups x OAD (*p* < 0.0001) (Figure 2F). For the DBP of the Hip-OAD category, there were significant differences between the Low-SMM/High-WC group and the Ref. group (70 and 95%CI [66; 74] vs. 73 [70; 76 mmHg], *p* < 0.0001, *diff*. −3 mmHg) and the High-SMM/High-WC group and Ref. group (75 [73; 77] vs. 73 [70; 76 mmHg], *p* < 0.0001, *diff*. +2 mmHg), showing a significant interaction of the groups x OAD (*p* = 0.019) (Figure 2G). Similarly, for the DBP of the Knee-OAD category, there were significant differences between the Low-SMM/High-WC group and the Ref. group (72 [68; 76] vs. 76 [73; 78 mmHg], *p* < 0.0001, *diff*. −4 mmHg) and the High-SMM/High-WC group and the Ref. group (75 [73; 76] vs. 76 [73; 78 mmHg], *p* < 0.0001, *diff*. +2 mmHg), showing a significant interaction of the groups x OAD (*p* < 0.0001) (Figure 2H).

### 3.3. Lipid Profile/Dyslipidaemia Markers (Secondary Outcomes)

**Figure 3:**
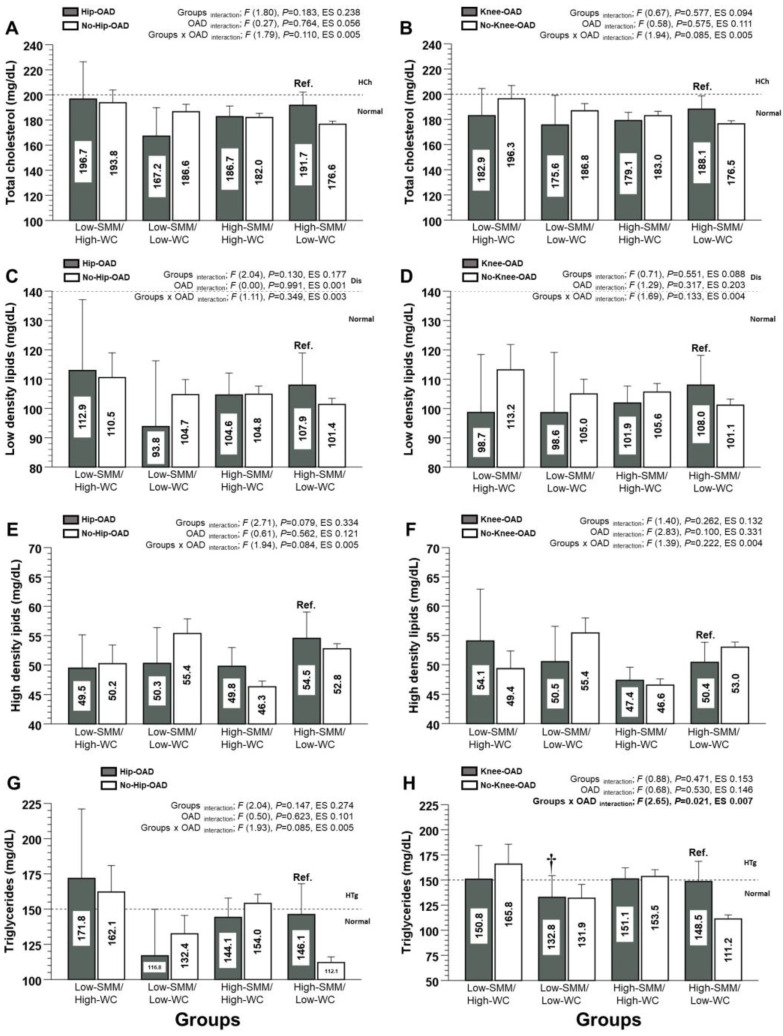
Lipid profile markers (total cholesterol (**A**,**B**), low-density lipids (**C**,**D**), high-density lipids (**E**,**F**), and triglycerides (**G**,**H**) for four different human adult body composition phenotypes (based on skeletal muscle mass using calf circumference and waist circumference) in relationship with hip and knee osteoarthritis diagnosed (OAD) categories). Groups are described as follows: (Low-SMM/High-WC) Low-skeletal muscle mass and high waist circumference phenotypical model. (Low-SMM/Low-WC) Low skeletal muscle mass and low waist circumference phenotypical model. (High-SMM/High-WC) High skeletal muscle mass and high waist circumference phenotypical model. (High-SMM/Low-WC) High skeletal muscle mass and low waist circumference phenotypical model. (Ref.) Reference group. Categories are described as follows: (Hip-OAD) Hip osteoarthritis diagnosed. (No-Hip-OAD) No hip osteoarthritis diagnosed. (Knee-OAD) Knee osteoarthritis diagnosed. (No-Knee-OAD) No Knee osteoarthritis diagnosed. (Ref.) Reference group. (ES) Denotes Cohen *d* effect size. (HCh) Hypercholesterolemia. (HTg) Hypertriglyceridemia. (†) Denotes significant differences vs. Ref. group at *p* < 0.05.

For the Tc, LDL-c, HDL-c, and Tg of the Hip-OAD category, there were no significant interaction groups x OAD (Figure 3A,C,G). Similarly, for the Tc, LDL-c, and HDL-c of the Knee-OAD category, there were no significant interactions for groups, OAD, and groups x OAD (Figure 3B,D,F). For the Tg of the Knee-OAD category, there was a significant difference between the Low-SMM/High-WC group and the Ref. group (150.8 and 95%CI [115; 186.0] vs. 148.5 [129.5; 167.5 mg/dL], *p* < 0.0001, *diff*. +2.3 mg/dL), showing a significant interaction for groups x OAD (*p* = 0.021) (Figure 3H).

### 3.4. Mineral Content, Non-Alcoholic Fatty Liver Disease, and Inflammation Markers (Secondary Outcomes)

**Figure 4:**
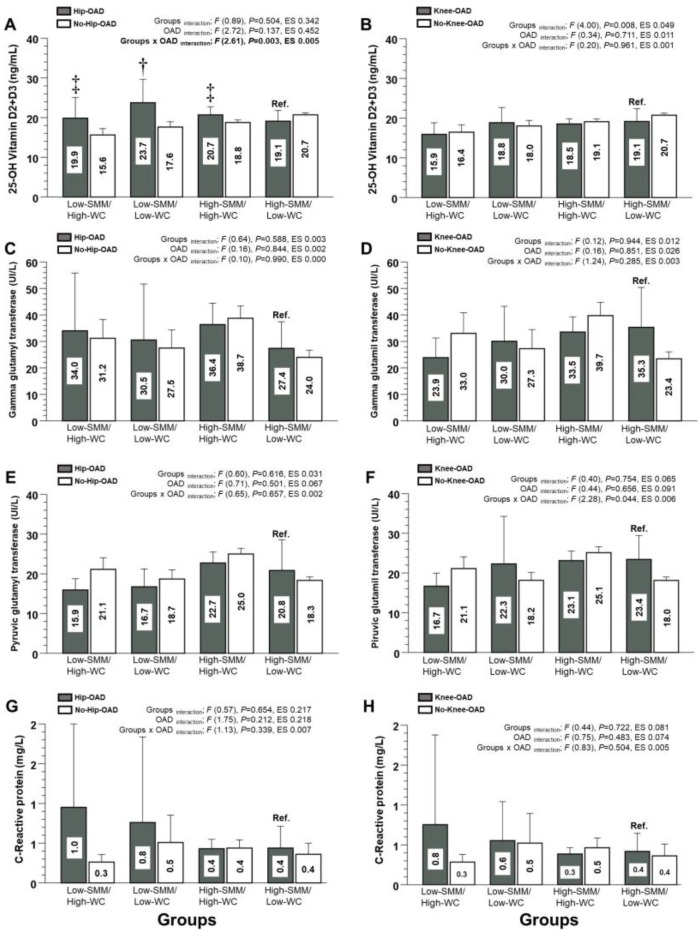
Markers of mineral content (**A**,**B**), non-alcoholic fatty liver disease (**C**–**F**), and inflammation (**G**,**H**) in adults for four different human adult body composition phenotypes (based on skeletal muscle mass using calf circumference and waist circumference) in relation to hip and knee osteoarthritis diagnosed (OAD) categories. Groups are described as follows: (Low-SMM/High-WC) Low skeletal muscle mass and high waist circumference phenotypical model. (Low-SMM/Low-WC) Low skeletal muscle mass and low waist circumference phenotypical model. (High-SMM/High-WC) High skeletal muscle mass and high waist circumference phenotypical model. (High-SMM/Low-WC) High skeletal muscle mass and low waist circumference phenotypical model. (Ref.) Reference group. Categories are described as follows: (Hip-OAD) Hip osteoarthritis diagnosed. (No-Hip-OAD) No hip osteo-arthritis diagnosed. (Knee-OAD) Knee osteoarthritis diagnosed. (No-Knee-OAD) No knee osteoarthritis diagnosed. (ES) Denotes Cohen *d* effect size. (‡) Denotes significant differences vs. Ref. group at *p* < 0.0001. (†) Denotes significant differences vs. Ref. group at *p* < 0.05.

For the vitamin D2 and D3 of the Hip-OAD category, there were significant differences between the Low-SMM/High-WC group and Ref. group (19.9 and 95%CI [12.2; 19.5] vs. 19.1 [16.3; 21.9 ng/mL], *p* < 0.0001, *diff*. +0.8 mg/dL), the Low-SMM/Low-WC group and Ref. group (23.7 [15.2; 22.4] vs. 19.1 [16.3; 21.9 ng/mL], *p* < 0.001, *diff*. +4.6 ng/mL), and the High-SMM/High-WC group and Ref. group (20.7 [17.2; 19.8] vs. 19.1 [16.3; 21.9 ng/mL], *p* < 0.0001, *diff*. +1.6 ng/mL) (Figure 4A), showing a significant interaction of groups x OAD (*p* = 0.021) (Figure 4A). On the other hand, for the vitamin D2 and D3 of the Knee-OAD category, as well as for the other outcomes (GGT, PGT, and C-Reactive protein), there were no significant interactions (Figure 4B).

### 3.5. Muscleness and Fatness Phenotypes for Predicting Plasma Glucose, and Blood Pressure Control

In comparison with the Ref. model (i.e., High-SMM/Low-WC), multinominal logistic regression reported that each Low-SMM/High-WC (β 1.055, OR 2.87 [1.80; 4.56], *p* < 0.0001), Low-SMM/Low-WC (β 0.662, OR 1.93 [1.26; 2.98], *p* = 0.003), and High-SMM/High-WC (β 0.882, OR 2.41 [1.69; 3.45], *p* < 0.0001) model showed a significant association with the suspect of ‘diabetes’, and the suspect of ‘HTN’ Low-SMM/High-WC (β 0.994, OR 2.70 [1.74; 4.19], *p* < 0.0001), Low-SMM/Low-WC (β 0.341, OR [1.00; 1.96], *p* = 0.046), and High-SMM/High-WC (β 1.139, OR 3.12 [2.46; 3.95], *p* < 0.0001) (Table 2).

Reporting by order, the risk (i.e., using the OR] for suffering from diabetes was higher firstly in the Low-SMM/High-WC model (OR 2.87 [1.80; 5.56]), secondly in the High-SMM/High-WC (OR 2.41 [1.69; 3.45]), and finally in the Low-SMM/Low-WC model (OR 1.93 [1.26; 2.98]) (Table 2). Similarly, the risk for suffering of ‘HTN’ was higher firstly in the High-SMM/High-WC model (OR 3.12 [2.46; 3.95]), secondly in the Low-SMM/High-WC model (OR 2.70 [1.74; 4.19]), and finally in the Low-SMM/Low-WC model (OR 1.40 [1.00; 1.96]) (Table 2).

On the other hand, in comparison with the Ref. model, the Low-SMM/High-WC (β 1.514, OR 4.54 [2.74; 7.54], *p* < 0.0001) and High-SMM/High-WC models (β 1.953, OR 7.04 [5.18; 9.59], *p* < 0.0001) showed a significant association with the suspect of MetS, and the suspect of moderate CVR Low-SMM/High-WC (β 1.016, OR 2.76 [1.26; 6.04], *p* = 0.011), and High-SMM/High-WC (β 0.629, OR 1.87 [1.16; 6.04], *p* = 0.009) (Table 2). Reporting by order, the OR for suffering from MetS was higher firstly in in High-SMM/High-WC (OR 7.04 [5.18; 9.59]), and secondly in Low-SMM/High-WC (OR 4.54 [2.74; 7.54]), (Table 2).

Finally, in comparison with the Ref. group, each Low-SMM/High-WC (β 1.711, OR 5.53 [2.78; 11.0], *p* < 0.0001), Low-SMM/Low-WC (β 0.873, OR 2.39 [1.53; 3.73], *p* < 0.0001), and High-SMM/High-WC model (β 1.279, OR 3.59 [2.37; 5.43], *p* < 0.0001) showed a significant association with the suspect of high CVR.

Reporting by order, the risk for suffering of high CVR was higher firstly in the Low-SMM/High-WC model (OR 5.53 [2.78; 11.0]), secondly in the High-SMM/High-WC model (OR 3.59 [2.37; 5.43]), and finally in the Low-SMM/Low-WC model (OR 2.39 [1.53; 3.73]) (Table 3).

## 4. Discussion

This study aimed to characterize cardiometabolic risk factors in hip and knee OAD across four phenotypes based on the CC (indicating SMM) and the WC (indicating adiposity). The key findings were that low SMM and high WC phenotypes showed markedly increased SBP and DBP versus the normal reference group (i.e., high SMM and low WC), irrespective of OAD status. These phenotypes also carried 2–3 fold higher OR for developing both diabetes and HTN. The highest risks for MetS and cardiovascular disease were seen mainly in the low SMM/high WC phenotype, but also the low SMM/low WC, and high SMM/high WC phenotypes modeled in the NHS16-17.

Notably, only the reference phenotype maintained a normal systolic blood pressure of approximately 116 mmHg, which was consistent with a previous meta-analysis showing that hypertension associates twice as strongly with radiographic knee OAD vs. controls [23]. Our findings indicate that SMM plays a key role in blood pressure regulation, as low SMM phenotypes had the greatest systolic elevations. Physiologically, an exercise-induced increase in SMM is associated with a 7 mmHg lower blood pressure [24]. A key implication is that an SMM loss elevates the systolic pressure in OAD, exacerbating the cardiometabolic risk, while exercise boosting the SMM can mitigate the risk and support cardiovascular health. Clinicians must consider managing blood pressure alongside OAD symptoms, as hypertension control is vital to prevent further cardiometabolic disease progression. Lifestyle modifications in diet and exercise could provide cardiovascular protection, which is an essential component of OAD care.

Lower extremity SMM is also independently associated with OAD onset [5], representing a possible early prevention target. Overall, these data demonstrate OAD to be a multifactorial condition requiring holistic management of muscle and fat composition and related cardiometabolic risk factors, not just joint symptoms. Optimizing physical activity and nutrition to preserve muscle mass, mitigate adiposity, and control blood pressure is critical for comprehensive OAD patient health promotion.

Skeletal muscle accounts for over 80% of glucose uptake [25]. Reduced muscle mass in OAD sarcopenia impairs glucose regulation, increasing diabetes, metabolic syndrome, and cardiovascular risk [26]. A meta-analysis showed OAD patients have double the metabolic syndrome prevalence versus controls despite 46% taking antihypertensives [8]. Only systolic pressure associated significantly with OAD prevalence after adjusting for geographical area, region, age, sex, and BMI, highlighting the hypertension–OAD link [27]. A longitudinal study also found 13% higher hypertension incidence in knee OAD [28], underscoring the need to investigate mechanisms linking blood pressure and metabolic OAD phenotypes. Altered biomarkers such as leptin and hsCRP likely contribute to obesity and inflammation in this population [3]. Sarcopenic muscle loss also causes oxidative stress, inflammation, and mitochondrial dysfunction, impairing glucose and fat metabolism [26]. However, we did not observe significant inflammation or fatty liver differences between the groups. Previous Chilean NHS research showed that a healthy lifestyle is associated with lower obesity/diabetes risk [29] and a meta-analysis revealed that exercise training improves liver enzymes in 4–12 weeks, [30], but physical inactivity increases OAD MetS and inflammation risk [31].

### Strengths and Limitations

The study limitations include that (i) despite using a representative Chilean cohort (n = 6233), only participants meeting the phenotype and OAD criteria were analyzed; (ii) data were collected 7 years ago; (iii) physical activity levels by intensity were self-reported via a questionnaire, potentially over/underestimating the true levels; (iv) as this was a cross-sectional study that associated outcomes, this study did not establish causality; and finally, (v) the ROC analysis revealed low sensitivity and specificity from WC in women. Regarding the ROC analyses, our test in WC revealed low sensitivity and specificity in women (sensitivity 52.4%, and specificity 0.05% to 80 cm as the cut-off point); however, these are the cut-off points for WC established by the Ministry of Chile for their NHS16-17 representative instrument. Therefore, we propose that other cut-off points of 102 cm for men (sensitivity 98.5% and specificity 66.8%) and 88 cm for women (sensitivity 85.4% and specificity 19.1%) could balance both the sensitivity and the specificity for future studies based on the NHS16–17. Additionally, we used the previously used cut-off points from [10] for CC and WC, which gave good results in a Latin-American population. On the other hand, some strengths of our study were (i) the nationally representative sampling of the NHS16–17; (ii) the novel epidemiological characterization of four muscle/fatness phenotypes in OAD vs. non-OAD groups, which have been rarely examined previously, but independent of their lifestyle are commonly expressed in the adult population; and (iii) the NHS16–17 is an open-access dataset from the Chilean Health Ministry available for future studies and comparisons with other countries.

## 5. Conclusions

In conclusion, hip/knee OAD was associated with elevated blood pressure and an OR above 2 for hypertension, MetS, and cardiovascular risks. OAD interacts substantially with cardiometabolic factors requiring holistic management beyond joint symptoms. Lifestyle optimization of physical activity and nutrition to preserve muscle mass, mitigate adiposity, and control blood pressure is essential for comprehensive OAD health promotion. 

## Figures and Tables

**Table 1:** Characteristics of an adult population with and without hip and knee osteoarthritis diagnosed and with low skeletal muscle mass plus high waist circumference, or with low skeletal muscle mass plus low waist circumference, based on the Chilean National Health Survey 2016–2017.

	Low-SMM/High-WC				Low-SMM/Low-WC			
Outcomes	Hip-OAD	No-Hip-OAD	Knee-OAD	No-Knee-OAD	Hip-OAD	No-Hip-OAD	Knee-OAD	No-Knee-OAD
Age (y)	76.9 ± 9.1	71.8 ± 8.3	73.4 ± 9.0	72.3 ± 8.5	76.9 ± 9.1	73.9 ± 9.2	74.7 ± 8.6	73.9 ± 9.2
Height (cm)	149.9 ± 6.8	152.9 ± 9.2	151.5 ± 7.4	152.8 ± 9.1	149.2 ± 6.2	151.2 ± 8.0	148.4 ± 5.3	151.8 ± 8.0
Weight (kg)	63.7 ± 8.7	66.4 ± 11.2	63.2 ± 8.9	66.4 ± 10.8	53.5 ± 5.3	53.2 ± 7.6	53.3 ± 6.3	53.3 ± 7.3
Body mass index (kg/m^2^)	28.4 ± 3.8	28.4 ± 3.7	27.7 ± 3.6	28.5 ± 3.7	24.4 ± 2.3	23.3 ± 2.9	24.3 ± 2.1	
Waist circumference (cm)	97.1 ± 9.1	98.0 ± 7.8	95.0 ± 4.8	98.2 ± 8.0	82.1 ± 5.1	78.9 ± 8.4	82.3 ± 6.6	78.8 ± 8.2
Calf circumference (cm)	29.2 ± 2.8	30.1 ± 2.5	29.2 ± 3.3	30.1 ± 2.3	29.5 ± 2.9	29.2 ± 2.6	29.8 ± 2.2	29.2 ± 2.6
Fasting plasma glucose (mg/dL)	98.8 ± 29.1	122.1 ± 68.9	114.3 ± 41.5	119.3 ± 68.9	104.4 ± 62.9	101.1 ± 39.2	104.3 ± 52.1	100.5 ± 39.3
HbA1c (%)	5.8 ± 0.5	7.2 ± 2.2	6.5 ± 1.0	7.2 ± 2.3	7.0 ± 2.7	6.6 ± 1.9	6.8 ± 2.1	6.6 ± 2.0
Systolic blood pressure (mmHg)	145 ± 17	147 ± 28	141 ± 21	147 ± 28	144 ± 22	140 ± 24	134 ± 25	140 ± 23
Diastolic blood pressure (mmHg)	70 ± 10	76 ± 11	72 ± 11	75 ± 11	73 ± 9	71 ± 11	71 ± 9	71 ± 10
Total cholesterol (mg/dL)	196.7 ± 49.2	193.8 ± 47.3	182.9 ± 40.5	196.3 ± 48.5	167.2 ± 33.7	186.6 ± 38.5	175.5 ± 51.7	186.8 ± 36.2
Low-density lipids (mg/dL)	112.9 ± 40.0	110.5 ± 39.2	98.7 ± 37.0	113.2 ± 39.3	93.8 ± 33.4	104.7 ± 33.6	98.6 ± 45.0	104.9 ± 31.6
High-density lipids (mg/dL)	49.5 ± 9.4	50.2 ± 14.8	54.1 ± 16.6	49.4 ± 13.7	50.3 ± 9.1	55.4 ± 16.3	50.5 ± 13.2	55.4 ± 16.1
Triglycerides (mg/dL)	171.8 ± 81.5	162.1 ± 287.8	150.8 ± 63.0	165.8 ± 90.6	116.8 ± 49.2	132.4 ± 85.0	132.7 ± 47.5	131.8 ± 87.0
25-OH Vitamin D2 + D3 (ng/mL)	19.8 ± 10.4	15.6 ± 7.6	15.9 ± 6.3	16.4 ± 8.6	23.7 ± 10.2	17.6 ± 8.7	18.8 ± 8.3	18.0 ± 9.0
Gamma glutamyl transaminase (UI/L)	34.0 ± 36.1	31.2 ± 33.0	23.9 ± 14.0	33.0 ± 35.6	30.5 ± 31.5	27.5 ± 44.6	30.0 ± 29.0	27.2 ± 45.5
Pyruvic glutamyl transaminase (UI/L)	15.9 ± 4.7	21.1 ± 13.4	16.7 ± 5.9	21.1 ± 13.5	16.7 ± 6.7	18.7 ± 15.2	22.2 ± 26.3	18.1 ± 12.5
C-Reactive protein (mg/L)	0.95 ± 2.17	0.26 ± 0.28	0.75 ± 1.95	0.28 ± 0.27	0.76 ± 1.1	0.51 ± 1.31	0.55 ± 0.84	0.52 ± 1.36
PA_VI_ (min/week)	I.A.P	8.0 ± 13.0	I.A.P	7.5 ± 15.0	I.A.P	1.3 ± 3.5	I.A.P	1.4 ± 3.7
PA_MI_ (min/week)	I.A.P.	4.4 ± 10.0	I.A.P	4.6 ± 10.2	10.0 ± 17.3	4.3 ± 8.6	15.0 ± 21.2	4.6 ± 9.2
PA_LI_ (min/week)	13.5 ± 14.3	14.5 ± 15.3	11.4 ± 17.3	14.8 ± 14.6	19.3 ± 14.3	12.4 ± 13.2	15.5 ± 11.3	12.5 ± 13.4

Data are shown as mean and ±SD. Groups are described as follows: (Low-SMM/High-WC) Low skeletal muscle mass plus high waist circumference. (Low-SMM/Low-WC) Low skeletal muscle mass plus low waist circumference. Categories of osteoarthritis conditions are described as follows: (Hip-OAD) hip osteoarthritis diagnosed; (No-Hip-OAD) no hip osteoarthritis diagnosed; (Knee-OAD) knee osteoarthritis diagnosed; (No-Knee-OAD) no knee osteoarthritis diagnosed; (PA_VI_) physical activity of vigorous intensity; (PA_MI_) physical activity of moderate intensity; (PA_LI_) physical activity of light intensity; (HTN) arterial hypertension; (HbA1c) glycated hemoglobin. (I.A.P.) Insufficient amount of participants to carry out statistics.

**Table 2:** Characteristics of an adult population with and without hip and knee osteo-arthritis diagnosed and with high skeletal muscle mass plus high waist circum-ference, or with high skeletal muscle mass plus low waist circumference, based on the Chilean National Health Survey 2016–2017.

	High-SMM/High-WC				High-SMM/Low-WC			
Outcomes	Hip-OAD	No-Hip-OAD	Knee-OAD	No-Knee-OAD	Hip-OAD	No-Hip-OAD	Knee-OAD	No-Knee-OAD
Age (y)	72.1 ± 7.8	69.6 ± 7.3	71.5 ± 8.1	69.5 ± 7.2	62.4 ± 11.7	41.3 ± 18.3	62.7 ± 10.6	40.9 ± 18.2
Height (cm)	153.2 ± 8.9	158.1 ± 9.3	154.8 ± 9.0	158.6 ± 9.3	151.5 ± 6.0	156.6 ± 6.6	152.9 ± 6.0	157.0 ± 6.6
Weight (kg)	76.9 ± 13.6	78.0 ± 12.2	78.2 ± 13.1	77.9 ± 12.1	61.0 ± 8.8	61.1 ± 8.2	61.6 ± 7.9	61.0 ± 8.3
Body mass index (kg/m^2^)	32.7 ± 5.0	31.3 ± 4.7	32.8 ± 5.3	31.1 ± 4.6	26.6 ± 3.4	24.9 ± 3.1	26.5 ± 3.0	24.9 ± 3.1
Waist circumference (cm)	103.8 ± 10.5	103.1 ± 9.4	104.4 ± 10.3	103.0 ± 9.7	80.1 ± 10.8	80.0 ± 7.3	82.4 ± 8.8	79.8 ± 7.3
Calf circumference (cm)	37.0 ± 3.8	37.0 ± 3.5	37.3 ± 3.6	36.9 ± 3.6	35.0 ± 2.2	34.5 ± 4.1	34.9 ± 2.0	34.5 ± 4.3
Fasting plasma glucose (mg/dL)	110.5 ± 43.1	111.9 ± 41.5	111.0 ± 38.0	112.0 ± 42.6	92.6 ± 13.3	90.3 ± 25.8	96.6 ± 25.4	90.0 ± 25.5
HbA1c (%)	6.8 ± 1.8	6.6 ± 1.5	6.6 ± 1.5	6.6 ± 1.6	6.1 ± 1.0	6.0 ± 1.58	6.6 ± 1.5	6.0 ± 1.5
Systolic blood pressure (mmHg)	141 ± 22	144 ± 22.0	141 ± 21	144 ± 22.1	126 ± 19	116 ± 19	134 ± 22	115.8 ± 18.5
Diastolic blood pressure (mmHg)	75 ± 9	78 ± 10	75 ± 9	78 ± 10	73 ± 10	70 ± 10	76 ± 12	70 ± 9
Total cholesterol (mg/dL)	182.6 ± 40.0	182.0 ± 41.2	179.0 ± 37.5	182.9 ± 41.8	191.7 ± 29.1	176.6 ± 37.4	188.1 ± 38.8	176.4 ± 37.0
Low-density lipids (mg/dL)	104.5 ± 35.3	104.8 ± 34.7	101.8 ± 33.4	105.6 ± 35.2	107.9 ± 30.3	101.3 ± 31.4	108.0 ± 37.4	101.1 ± 31.0
High-density lipids (mg/dL)	49.7 ± 15.1	46.3 ± 12.3	47.3 ± 12.7	46.5 ± 12.8	54.5 ± 12.4	52.7 ± 13.1	50.4 ± 12.6	52.9 ± 13.1
Triglycerides (mg/dL)	144.1 ± 64.9	154.0 ± 80.9	151.0 ± 63.7	153.5 ± 82.2	146.1 ± 60.5	112.0 ± 61.4	148.5 ± 74.0	111.2 ± 60.1
25-OH Vitamin D2 + D3 (ng/mL)	20.6 ± 10.0	18.7 ± 8.2	18.5 ± 8.3	19.0 ± 8.6	19.0 ± 6.0	20.7 ± 8.3	19.1 ± 9.4	20.7 ± 8.2
Gamma glutamyl transaminase (UI/L)	36.3 ± 38.4	38.7 ± 58.5	33.5 ± 33.1	39.7 ± 60.5	27.3 ± 27.8	23.9 ± 41.1	35.3 ± 55.6	23.4 ± 39.6
Pyruvic glutamyl transaminase (UI/L)	22.7 ± 13.1	24.9 ± 17.8	23.1 ± 13.9	25.1 ± 18.0	20.8 ± 21.4	18.3 ± 13.3	23.4 ± 22.2	18.1 ± 12.9
C-Reactive protein (mg/L)	0.43 ± 0.46	0.43 ± 0.80	0.38 ± 0.41	0.46 ± 0.87	0.43 ± 0.64	0.35 ± 0.93	0.41 ± 0.64	0.36 ± 0.95
PA_VI_ (min/week)	6.5 ± 11.0	6.4 ± 12.2	6.8 ± 11.7	6.3 ± 12.2	3.7 ± 7.5	5.6 ± 11.8	0.2 ± 0.7	5.8 ± 11.9
PA_MI_ (min/week)	7.7 ± 12.4	5.5 ± 10.4	8.2 ± 12.4	5.2 ± 10.3	5.0 ± 10.8	3.8 ± 9.7	7.4 ± 13.8	3.7 ± 9.5
PA_LI_ (min/week)	14.7 ± 13.7	13.2 ± 14.0	14.8 ± 13.9	13.0 ± 13.9	13.1 ± 15.6	13.5 ± 14.1	12.8 ± 14.8	13.5 ± 14.1

Data are shown as mean and ±SD. Groups are described as follows: (Low-SMM/High-WC) Low skeletal muscle mass plus high waist circumference. (Low-SMM/Low-WC) Low skeletal muscle mass plus low waist circumference. Categories of osteoarthritis conditions are described as follows: (Hip-OAD) hip osteoarthritis diagnosed; (No-Hip-OAD) no hip osteoarthritis diagnosed; (Knee-OAD) knee osteoarthritis diagnosed; (No-Knee-OAD) no knee osteoarthritis diagnosed; (PA_VI_) physical activity of vigorous intensity; (PA_MI_) physical activity of moderate intensity; (PA_LI_) physical activity of light intensity; (HTN) arterial hypertension; (HbA1c) glycated hemoglobin. (N.R.)

**Table 3:** Multinominal logistic regression with odds ratios by each phenotype group and according to the risk for suffering different cardiometabolic conditions.

Outcomes	β	SE	Wald	McFadden Pseudo R^2^	OR (95%CI)	*p*-Value
Suspected of ‘Diabetes’						
Model 1: Low-SMM/High-WC	1.055	0.237	19.883	0.146	2.87 (1.80; 4.56)	*p* < 0.0001
Model 2: Low-SMM/Low-WC	0.662	0.220	9.076		1.93 (1.26; 2.98)	*p* = 0.003
Model 3: High-SMM/High-WC	0.882	0.182	23.488		2.41 (1.69; 3.45)	*p* < 0.0001
Model 4: High-SMM/Low-WC	-	-	-		1.00 (Ref.)	-
Suspected of ‘Arterial Hypertension’						
Model 1: Low-SMM/High-WC	0.994	0.224	19.703	0.322	2.70 (1.74; 4.19)	*p* < 0.0001
Model 2: Low-SMM/Low-WC	0.341	0.170	3.996		1.40 (1.00; 1.96)	*p* = 0.046
Model 3: High-SMM/High-WC	1.139	0.120	89.502		3.12 (2.46; 3.95)	*p* < 0.0001
Model 4: High-SMM/Low-WC	-	-	-		1.00 (Ref.)	-
Suspected of ‘Metabolic Syndrome’						
Model 1: Low-SMM/High-WC	1.514	0.258	34.342	0.237	4.54 (2.74; 7.54)	*p* < 0.0001
Model 2: Low-SMM/Low-WC	−0.113	0.211	0.285		0.89 (0.59; 1.35)	*p* = 0.594
Model 3: High-SMM/High-WC	1.953	0.157	154.583		7.04 (5.18; 9.59)	*p* < 0.0001
Model 4: High-SMM/Low-WC	-	-	-		1.00 (Ref.)	-
Suspected of ‘Moderate Cardiovascular Risk’						
Model 1: Low-SMM/High-WC	1.016	0.400	6.457	0.221	2.76 (1.26; 6.04)	*p* = 0.011
Model 2: Low-SMM/Low-WC	−0.076	0.306	0.062		0.92 (0.50; 1.68)	*p* = 0.803
Model 3: High-SMM/High-WC	0.629	0.241	6.798		1.87 (1.16; 3.01)	*p* = 0.009
Model 4: High-SMM/Low-WC	-	-	-		1.00 (Ref.)	-
Suspected of ‘High Cardiovascular Risk’						
Model 1: Low-SMM/High-WC	1.711	0.352	23.705	0.221	5.53 (2.78; 11.0)	*p* < 0.0001
Model 2: Low-SMM/Low-WC	0.873	0.227	14.766		2.39 (1.53; 3.73)	*p* < 0.0001
Model 3: High-SMM/High-WC	1.279	0.211	36.566		3.59 (2.37; 5.43)	*p* < 0.0001
Model 4: High-SMM/Low-WC	-	-	-		1.00 (Ref.)	-

Data are shown as mean and 95% CI for odds ratios (OR). Groups are described as follows: (Low-SMM/High-WC) Low skeletal muscle mass and high waist circumference phenotypical model. (Low-SMM/Low-WC) Low skeletal muscle mass and low waist circumference phenotypical model. (High-SMM/High-WC) High skeletal muscle mass and high waist circumference phenotypical model. (High-SMM/Low-WC) High skeletal muscle mass and low waist circumference phenotypical model. (SMM) Skeletal muscle mass. (WC) Waist circumference. (Model 1) Include calf circumference [CC] ≤ 33.9 cm and WC ≥ 90.0 cm for men, or CC ≤ 32.9 cm and WC ≥ 80.0 cm, and geographic area, region, age, body mass index, and sex. (Model 2) Include calf circumference [CC] ≤ 33.9 cm and WC ≤ 89.9 cm for men, or CC ≤ 32.9 cm and WC ≤ 79.9 cm for women, and geographic area, region, age, body mass index, and sex. (Model 3) Include calf circumference [CC] ≥ 34.0 cm and WC ≥ 90.0 cm for men, or CC ≥ 33.0 cm and WC ≥ 80.0 cm for women, and geographic area, region, age, body mass index, and sex. (Model 4) Include calf circumference [CC] ≥ 34.0 cm and WC ≤ 89.9 cm for men, or CC ≥ 33.0 cm and WC ≤ 79.9 cm for women, and geographic area, region, age, body mass index, and sex. (Ref.) Reference group. (HTN) Arterial hypertension. (MetS) Metabolic syndrome. (CVR) Cardiovascular risk.

## Data Availability

All data information can be found freely accessed at the Epidemiological Unit of the Chilean health Ministry at http://epi.minsal.cl/encuesta-ens-descargable (accessed on 3 July 2023).

## References

[1] Long H., Liu Q., Yin H., Wang K., Diao N., Zhang Y., Lin J., Guo A. (2022). Prevalence trends of site-specific osteoarthritis from 1990 to 2019: Findings from the Global Burden of Disease Study 2019. Arthritis Rheumatol..

[2] Allen K., Thoma L., Golightly Y. (2022). Epidemiology of osteoarthritis. Osteoarthr. Cartil..

[3] Dell’Isola A., Allan R., Smith S., Marreiros S., Steultjens M. (2016). Identification of clinical phenotypes in knee osteoarthritis: A systematic review of the literature. BMC Musculoskelet Disord..

[4] Pegreffi F., Balestra A., De Lucia O., Smith L., Barbagallo M., Veronese N. (2023). Prevalence of sarcopenia in knee osteoarthritis: A systematic review and meta-analysis. J. Clin. Med..

[5] Lee S.Y., Ro H.J., Chung S.G., Kang S.H., Seo K.M., Kim D.-K. (2016). Low skeletal muscle mass in the lower limbs is independently associated to knee osteoarthritis. PLoS ONE.

[6] Abramoff B., Caldera F.E. (2020). Osteoarthritis: Pathology, diagnosis, and treatment options. Med. Clin..

[7] Valenzuela P.L., Carrera-Bastos P., Castillo-García A., Lieberman D.E., Santos-Lozano A., Lucia A. (2023). Obesity and the risk of cardiometabolic diseases. Nat. Rev. Cardiol..

[8] Mathieu S., Couderc M., Tournadre A., Soubrier M. (2019). Cardiovascular profile in osteoarthritis: A meta-analysis of cardiovascular events and risk factors. Jt. Bone Spine Rev. Du Rhum..

[9] Alvarez C., Campos-Jara C., Ciolac E.G., Vega-Guimaraes G., Andrade-Mayorga O., Cano-Montoya J., Andrade D.C., Delgado-Floody P., Alonso-Martínez A., Izquierdo M. (2023). Pacientes hipertensos muestran una mayor respuesta de la frecuencia cardíaca durante el ejercicio progre-sivo en relación con pares adultos normotensos: PROYECTO VASCU-HEALTH (Hypertensive patients show higher heart rate response during incremental exercise and elevated arterial age estimation than normotensive adult peers: VASCU-HEALTH PROJECT). Retos.

[10] Pagotto V., dos Santos K.F., Malaquias S.G., Bachion M.M., Silveira E.A. (2018). Calf circumference: Clinical validation for evaluation of muscle mass in the elderly. Rev. Bras. De Enferm..

[11] Pieńkowska J., Brzeska B., Kaszubowski M., Kozak O., Jankowska A., Szurowska E. (2020). The correlation between the MRI-evaluated ectopic fat accumulation and the incidence of diabetes mellitus and hypertension depends on body mass index and waist circumference ratio. PLoS ONE.

[12] Dégano I.R., Marrugat J., Grau M., Salvador-González B., Ramos R., Zamora A., Martí R., Elosua R. (2017). The association between education and cardiovascular disease incidence is mediated by hypertension, diabetes, and body mass index. Sci. Rep..

[13] Wåhlin-Larsson B., Wilkinson D.J., Strandberg E., Hosford-Donovan A., Atherton P.J., Kadi F. (2018). Mechanistic links underlying the impact of C-reactive protein on muscle mass in elderly. Cell. Physiol. Biochem..

[14] Cigarroa I., Zapata-Lamana R., Leiva-Gajardo G., Vasquez E., Parrado-Romero E., Vásquez-Gomez J., Álvarez C., Petermann-Rocha F., Reyes-Molina D. (2022). Características de la adherencia y motivos del abandono de las intervenciones basadas en el ejercicio físico en adultos mayores en América Latina: Una revisión de alcance (Adherence characteristics and reasons for abandonment of physical exercise-based in: Una revisión de alcance. Retos.

[15] Rolland Y., Lauwers-Cances V., Cournot M., Nourhashémi F., Reynish W., Rivière D. (2003). Sarcopenia, calf circumference, and physical function of elderly women: A cross-sectional study. J. Am. Geriatr. Soc..

[16] Delgado-Floody P., Lepin C.G., Ramirez R., Fuentes C.M., Saavedra P.I., Campos C., Cristi-Montero C., Sotomayor E.M., Caparrós C. (2023). Lifestyle and cardiometabolic risk factors in the ethnic and non-ethnic population> 15 years of age: Results from the National Chilean Health Survey 2016–2017. Nutr. Hosp..

[17] Whelton P.K., Carey R.M., Aronow W.S., Casey D.E., Collins K.J., Himmelfarb C.D., DePalma S.M., Gidding S., Jamerson K.A., Jones D.W. (2018). 2017 ACC/AHA/AAPA/ABC/ACPM/AGS/APhA/ASH/ASPC/NMA/PCNA Guideline for the Prevention, Detection, Evaluation, and Management of High Blood Pressure in Adults: Executive Summary: A Report of the American College of Cardiology/American Heart Association Task Force on Clinical Practice Guidelines. Hypertension.

[18] Petermann F., Duran E., Labraña A.M., Martínez M.A., Leiva A.M., Garrido-Mendez A., Poblete-Valderrama F., Díaz-Martínez X., Salas C., Celis-Morales C. (2017). Risk factors associated with hypertension. Analysis of the 2009–2010 Chilean health survey. Rev. Medica Chile.

[19] NCEP (2002). Third Report of the National Cholesterol Education Program (NCEP) Expert Panel on Detection, Evaluation, and Treatment of High Blood Cholesterol in Adults (Adult Treatment Panel III) Final Report. Circulation.

[20] Concha-Cisternas Y., Vásquez-Gómez J., Castro-Piñero J., Petermann-Rocha F., Parra-Soto S., Matus-Castillo C., Garrido-Méndez Á., Poblete-Valderrama F., Celis-Morales C. (2023). Niveles de actividad física y tiempo sedente en personas mayores con fragilidad: Resultados de la Encuesta Nacional de Salud 2016–2017. Nutr. Hosp..

[21] WHO (2000). Obesity: Preventing and Managing the Global Epidemic.

[22] MINSAL (2018). Informe de Encuesta Nacional de Salud 2016–2017.

[23] Lo K., Au M., Ni J., Wen C. (2022). Association between hypertension and osteoarthritis: A systematic review and meta-analysis of observational studies. J. Orthop. Transl..

[24] Hansen A.H., Nielsen J.J., Saltin B., Hellsten Y. (2010). Exercise training normalizes skeletal muscle vascular endothelial growth factor levels in patients with essential hypertension. J. Hypertens..

[25] DeFronzo R.A., Ferrannini E., Sato Y., Felig P., Wahren J. (1981). Synergistic interaction between exercise and insulin on peripheral glucose uptake. J. Clin. Investig..

[26] Rubio-Ruiz M.E., Guarner-Lans V., Pérez-Torres I., Soto M.E. (2019). Mechanisms underlying metabolic syndrome-related sarcopenia and possible therapeutic measures. Int. J. Mol. Sci..

[27] Niu J., Clancy M., Aliabadi P., Vasan R., Felson D.T. (2017). Metabolic syndrome, its components, and knee osteoarthritis: The Framingham Osteoarthritis Study. Arthritis Rheumatol..

[28] Veronese N., Stubbs B., Solmi M., Smith T.O., Noale M., Schofield P., Maggi P. (2018). Knee osteoarthritis and risk of hypertension: A longitudinal cohort study. Rejuvenation Res..

[29] Leiva A.M., Petermann-Rocha F., Martínez-Sanguinetti M.A., Troncoso-Pantoja C., Concha Y., Garrido-Méndez A., Díaz-martínez X., Lanuza-Rilling F., Ulloa N., Martorell M. (2018). Asociación de un índice de estilos de vida saludable con factores de riesgo cardiovascular en población chilena. Rev. Medica De Chile.

[30] dos Santos L.L., Pinto de Castro J.B., Gama Linhares D., Barros dos Santos A.O., de Souza Cordeiro L., Borba-Pinheiro C.J., Gómez de souza V. (2023). Efectos del ejercicio físico sobre los biomarcadores hepáticos en adultos: Revisión sistemática y metanálisis (Effects of Physical Exercise on Hepatic Biomarkers in Adult Individuals: A Systematic Review and Meta-Analysis). Retos.

[31] Shin D. (2014). Association between metabolic syndrome, radiographic knee osteoarthritis, and intensity of knee pain: Results of a national survey. J. Clin. Endocrinol. Metab..

